# Fecal microbiota transplantation from patients with rheumatoid arthritis causes depression-like behaviors in mice through abnormal T cells activation

**DOI:** 10.1038/s41398-022-01993-z

**Published:** 2022-06-01

**Authors:** Yaoyu Pu, Qiuping Zhang, Zhigang Tang, Chenyang Lu, Liang Wu, Yutong Zhong, Yuehong Chen, Kenji Hashimoto, Yubin Luo, Yi Liu

**Affiliations:** 1 Department of Rheumatology and Immunology, West China Hospital, Sichuan University, Chengdu, Sichuan 610041 China; 2 Division of Clinical Neuroscience, Chiba University Center for Forensic Mental Health, Chiba, 260-8670 Japan

**Keywords:** Depression, Diagnostic markers

## Abstract

Depression is common in patients with rheumatoid arthritis (RA); however, the precise mechanisms underlying a link between depression and RA remain unclear. Accumulating evidence suggests the role of gut–microbiota–brain axis in depression. In this study, we investigated whether collagen-induced arthritis (CIA) mice produce depression-like behaviors and abnormal composition of gut microbiota. Furthermore, we investigated whether fecal microbiota transplantation (FMT) from RA patients causes depression-like phenotypes in antibiotic cocktail (ABX)-treated mice. CIA mice displayed depression-like behaviors, increased blood levels of pro-inflammatory cytokine interleukin-6 (IL-6), decreased expression of synaptic proteins in the prefrontal cortex (PFC), and abnormal composition of gut microbiota. Furthermore, FMT from RA patients caused depression-like phenotypes, alterations of gut microbiota composition, increased levels of IL-6 and tumor necrosis factor-α (TNF-α), and downregulation of synaptic proteins in the PFC compared to FMT from healthy controls. There were correlations between relative abundance of microbiota and plasma cytokines, expression of synaptic proteins in the PFC or depression-like behaviors. Interestingly, FMT from RA patients induced T cells differentiation in Peyer’s patches and spleen. Reduced percentage of Treg cells with an increase of Th1/Th2 index was observed in the mice after FMT from RA patients. These findings suggest that CIA mice exhibit depression-like behaviors, systemic inflammation, and abnormal composition of gut microbiota, and that FMT from RA patients produces depression-like behaviors in ABX-treated mice via T cells differentiation. Therefore, abnormalities in gut microbiota in RA patients may contribute to depression via gut–microbiota–brain axis.

## Introduction

Rheumatoid arthritis (RA) is a systemic, chronic autoimmune disease characterized by joint pain, swelling, and damage [1]. RA and major depressive disorder (MDD) usually occur together [2–4]. Prevalence of MDD in RA patients vary from 13% to 42%, two to four times higher than the prevalence in the general population [5]. Complications of depression reduce the treatment response of RA patients and increase the patients’ mortality [6]. Although the precise mechanisms underlying the high occurrence of depression in RA patients are unknown, abnormalities in gut microbiota composition may play a crucial role in link between RA and depression [7].

Clinical findings suggest that the composition of gut microbiota was altered in RA patients before the appearance of typical joint symptoms [8–10]. Although antibiotics were not used in the treatment of RA after the 1950s, antibiotics did have a certain therapeutic effect for RA patients [11]. In collagen-induced arthritis (CIA) mice, which is widely used as an animal model of RA, microbial depletion could reduce the degree of joint swelling and the severity of the disease [12], suggesting a role of gut microbiota in RA. Furthermore, RA patients have similar abnormal composition of gut microbiota in MDD patients [13]. However, it is currently unclear whether abnormal composition of gut microbiota causes high prevalence of depression in RA.

The brain–gut–microbiota axis is a complex, two-way signaling system that plays a central role in depression [14, 15]. Accumulating evidence suggests that MDD patients have an abnormal composition of gut microbiota compared to healthy control subjects [16–18], and that the symptoms of depression could be improved after the microbiota changed [19]. Previously, we reported that abnormal composition of gut microbiota might contribute to depression-like behaviors in rodents [20–24]. Collectively, it is possible that alterations in the gut microbiota play a crucial role in depressive symptoms in MDD patients via the brain–gut–microbiota axis.

Peyer’s patches (PPs), a gut-associated lymphoid tissue, can receive signal molecules from gut bacteria and differentiate naïve T cells into regulatory T (Treg) cells or T helper (Th) cells [25]. Several reports have shown that Treg cells are reduced in patients with MDD, and that the use of antidepressants ameliorated this reduction [26–29]. In addition, the increased index of Th1/Th2 cells has been shown in patients with MDD [30]. However, it is largely unknown whether abnormal composition of gut microbiota activates the T cells differentiation, resulting in depression.

The current study has three main purposes. First, we examined whether CIA mice exhibit depression-like behaviors and abnormal composition of gut microbiota. Second, we investigated whether the fecal microbiota transplantation (FMT) from RA patients can produce depression-like behaviors and abnormal composition of gut microbiota in antibiotic cocktail (ABX)-treated mice. Furthermore, we examined correlations between relative abundance of microbiota and behavioral data, blood levels of pro-inflammatory cytokines, or synaptic proteins in the brain. Finally, we performed the flow cytometry to analysis the changes in the proportion and composition of T cells and the correlations between gut microbiota.

## Materials and methods

### Animals

Male DBA/1 J mice (8 weeks old, 20–25 g) and C57BL/6 mice (8 weeks old, 20–25 g) were purchased from HuaFuKang Co., Ltd. (Beijing, China). Mice were housed in controlled temperature (23 ± 1 °C) and 12-h light/dark cycles (lights on between 07:00–19:00) with libitum food (SPF mice food; Beijing Keao Xieli Feed Co., Ltd., Beijing, China) and water. The experimental protocol of this study was approved by the Sichuan University Institutional Animal Care and Use Committee (Permission number 20211189 A). The animals were deeply anesthetized with isoflurane and rapidly killed by cervical dislocation. All efforts were made to minimize mice suffering. The sample size was chosen as reported previously.

### Collagen-induced arthritis (CIA) model

**Fig. 1:**
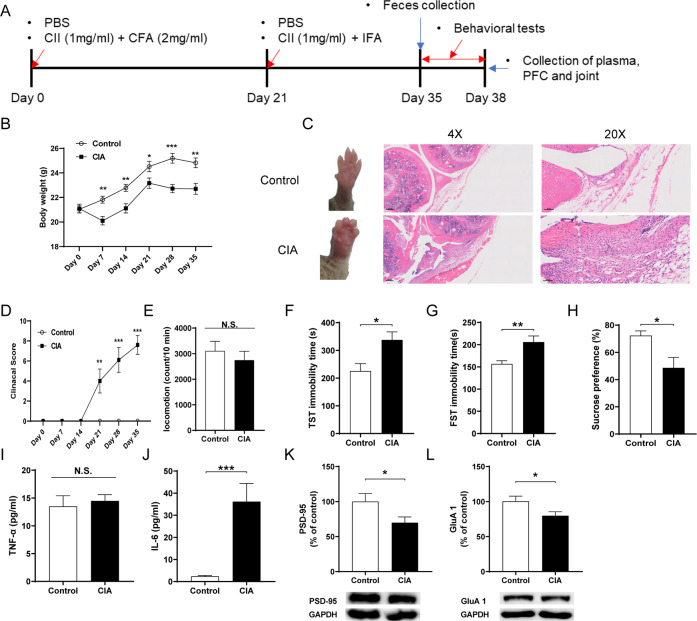
Joint swelling and depression-like phenotypes after collagen-induced arthritis. **A** Schedule of treatment, behavioral tests and sample collection. **B** Body weight among two groups (repeated measure one-way ANOVA, F_1,18_ = 22.988. *P* < 0.001). **C** Representative images of wrist joint and representative H&E staining on the knee joint. **D** Clinical score (repeated measure one-way ANOVA, F_1,18_ = 34.892. *P* < 0.001). **E** Locomotion test (LMT) (Student *t*-test: *t* = 0.701, *P* = 0.492). **F** TST (Student *t*-test: *t* = −2.843, *P* = 0.011). **G** FST (Student *t*-test: *t* = −3.225, *P* = 0.005). **H** SPT (Student *t*-test: *t* = 2.831, *P* = 0.011). **I** Plasma TNF-α levels (Student *t*-test: *t* = −0.446, *P* = 0.661). **J** Plasma IL-6 levels (Student *t*-test: *t* = −4.127, *P* < 0.001). **K** PSD-95 (Student *t*-test: *t* = 2.138, *P* = 0.046). **L** GluA1 (Student *t*-test: *t* = 2.204, *P* = 0.045). Data are shown as mean ± S.E.M. (*n* = 10). *P < 0.05, **P < 0.01, ***P < 0.001.

CIA was performed using the method of previous report [31]. Male DBA/1 J mice (8 weeks) were randomly divided the two groups, and immunized intradermally at 1.5 cm from the base of the tail with 100 μl mixed liquids of bovine type II collagen (CII) (Cat number: 20022, Chondrex, WA, USA) and an equal volume of Complete Freund’s Adjuvant (CFA) (Cat number: 7001, WA, USA). After 21 days, mice were boosted with 100 μl CII blend with the same amount of incomplete Freund’s adjuvant (IFA) (Cat number: 7002, Chondrex, WA, USA). The clinical scores 0–4 of arthritis were evaluated from day 21 to 35 after the first immunization. Briefly, 0, paws with no swelling; (1) erythema or swelling of finger joints; (2) mild swelling of paws wrist or ankle joints; (3) whole paws with severe swelling; (4) paws with deformity or ankylosis. Each paw score was added and the highest score of per mouse is 16. The locomotion test and tail suspension test (TST) were performed on day 36; TST was performed 2 h after the locomotion test. The forced swimming test (FST) and sucrose preference test (SPT) were performed on day 37 and day 38, respectively. Plasma, joint and prefrontal cortex (PFC) were collected on day 39 (Fig. 1A).

### Behavioral tests for depression-like phenotypes

Behavioral tests such as locomotion test (LMT), tail suspension test (TST), forced swimming test (FST), and 1% sucrose preference test (SPT), were performed as reported previously [20]. All behavioral tests were performed by experimenters who were blind to the treatment.

#### Locomotion test

The locomotor activity was measured by an animal movement analysis system EthoVision XT 12 (Noldus Information Technology, VA, USA). The experimental cages are 500 mm × 500 mm × 350 mm (length × width × height). The mouse was put in the cage and the total locomotor activities were counts for 10 min. Cages were cleaned between test sessions.

#### Tail suspension test

Stick a small piece of tape at around 2 cm from the tip of the mice tail. A single hoop was wrapped in the tape and mice were hung individually on a hook at 30 cm from the test bench. The immobility time of each mouse was recorded automatically by EthoVision XT 12 (Noldus Information Technology, VA, USA) for 10 min. Immobility time will be calculated only a mouse hung passively and remained completely motionless.

#### Forced swimming test

The immobility time of each mouse in FST was also mechanically documented by EthoVision XT 12 (Noldus Information Technology, VA, USA). The animals were placed in a cylinder (diameter: 15 cm; height 25 cm) containing 15 cm of water and the temperature was kept at 23 ± 1 °C. The immobility time was recorded and measured for a period of 6 min by the apparatus.

#### Sucrose preference test

Mice were exposed to water and 1% sucrose solution for 48 h, followed by 4 h of water and food deprivation. Then the animals were allowed to intake water and 1% sucrose solution freely for 1 h. The consumption of water and 1% sucrose solution during this 1 h of each mouse was calculated and displayed as the proportion of sucrose solution in total drinking consumptions.

### Collection of fecal samples from RA patients and mice

We collected fresh fecal samples from healthy controls (HCs, *n* = 7) and untreated RA patients (RAs, *n* = 6, Table S1). Diagnosis for RA was based on ACR (American College of Rheumatology)/EULAR (European League Against Rheumatism) 2010 rheumatoid arthritis classification criteria, and patients with the following conditions will be excluded: (1) have used antibiotics or biological agents or high-dose glucocorticoids in the past 3 months; (2) undergone gastrointestinal endoscopy, surgery in nearly 6 months; (3) special dietary habits, pregnant or lactating women; (4) suffered from severe infection and from other chronic diseases such as hypertension, diabetes, tumor, systemic lupus erythematosus (SLE) or so on. The fecal samples were divided and stored into sterilized screw cap microtubes at −80 °C immediately after defecation. After the collection of fecal samples, we mixed and crushed the frozen feces on dry ice separately from all RA patients or HCs, weighed 1 g into a 1.5 ml sterilized tube. Added 10 ml water for 1 g feces and mixed well, centrifuged at 3000 rpm/min for 5 min and extracted the supernatant for FMT as previously reported [20]. The study procedure was approved by the Biomedical Research Ethics Committee, West China Hospital of Sichuan University (ChiCTR1900022605), and the written consents were obtained from all the participants according to the Declaration of Helsinki. Sociodemographic factors and clinical symptoms are summarized in Table S1.

Mouse fresh fecal samples were collected at around 10:00 to avoid any circadian effects on the microbiome. The fecal samples were individually placed into sterilized screw cap microtubes immediately after defecation, as described above [20]. These samples were stored at −80 °C prior to 16S ribosome RNA sequencing.

### Antibiotic cocktail treatment, FMT, and behavioral tests

**Fig. 2:**
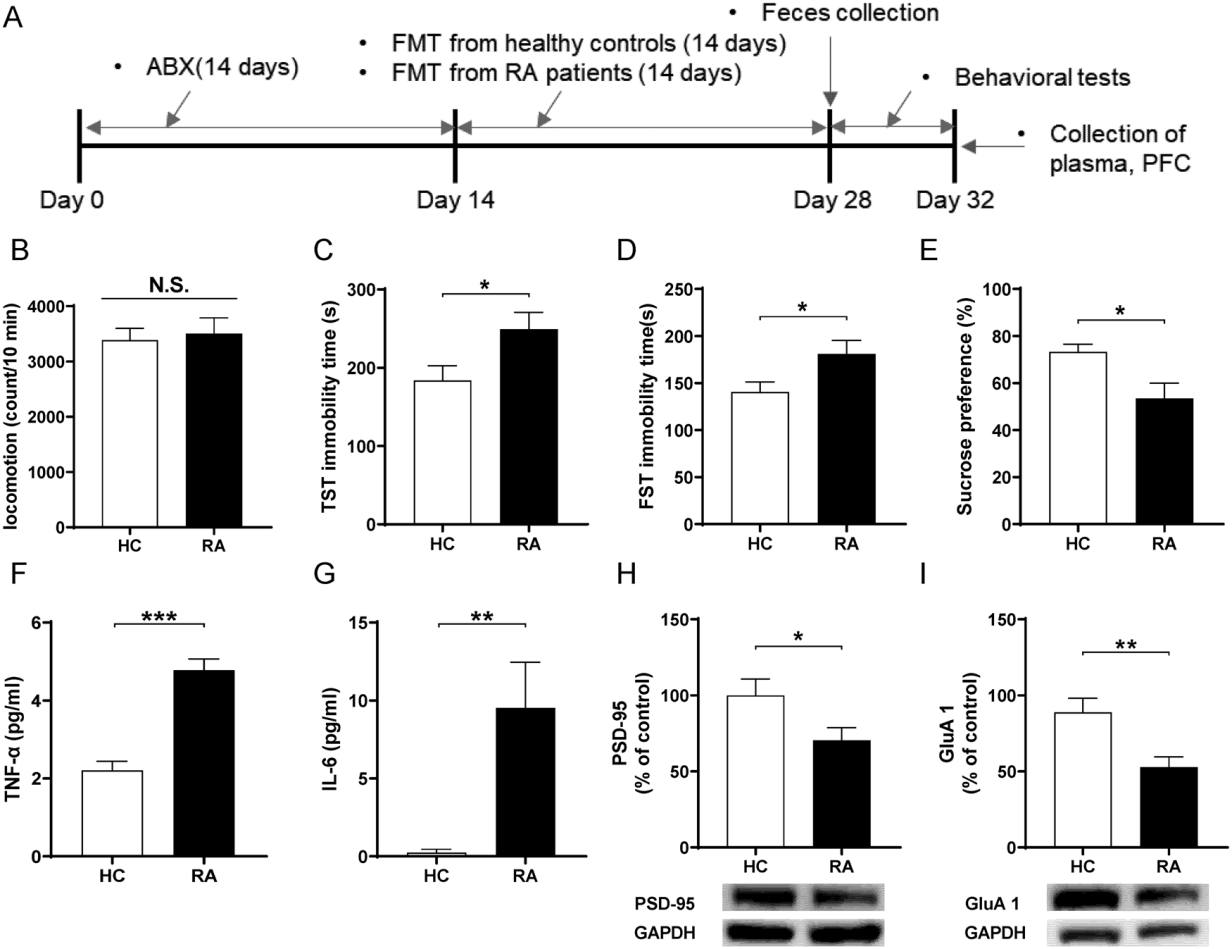
Effects of FMT from RA patients and healthy controls in ABX-treated mice. **A** Schedule of treatment, behavioral tests and sample collection. **B** LMT (Student *t*-test: *t* = −0.331, *P* = 0.744). **C** TST (Student *t*-test: *t* = −2.288, *P* = 0.034). **D** FST (Student *t*-test: *t* = −2.302, *P* = 0.033). **E** SPT (Student *t*-test: *t* = 2.738, *P* = 0.012). **F** Plasma TNF-α levels (Student *t*-test: *t* = −6.932, *P* < 0.001). **G** Plasma IL-6 levels (Student *t*-test: *t* = −3.156, *P* = 0.005). **H** PSD-95 (Student *t*-test: *t* = 2.174, *P* = 0.043). **I** GluA1 (Student *t*-test: *t* = 3.132, *P* = 0.006). Data are shown as mean ± S.E.M. (*n* = 10). *P < 0.05, **P < 0.01, ***P < 0.001.

The mice were randomly divided into the two groups. Broad-spectrum antibiotics (ABX: ampicillin 1 g/L, neomycin sulfate 1 g/L, metronidazole 1 g/L, Sigma-Aldrich Co. Ltd, St. Louis, MO, USA) dissolved in drinking water were given ad libitum to male C57BL/6 mice for 14 consecutive days (days 1–14) as previously reported [20, 22–24, 32]. The drinking solution was renewed every 2 days. Subsequently, the FMT from the HCs and RAs was performed for 14 days (day 15–day 28). The locomotion test and TST were performed on day 29; the TST was performed 2 h after the locomotion test. The FST and SPT were performed on day 30 and day 31, respectively. Plasma and PFC were collected on day 32 (Fig. 2A).

### Western blot analysis

Western blot analysis for synaptic proteins was performed as previously reported [20]. Basically, the PFC tissue was homogenized in Laemmli lysis buffer. 50 μg protein was calculated with a BCA protein assay kit (Beyotime, Shanghai, China) and heated for 5 min at 95 °C with a quarter volume of SDS-PAGA sample loading buffer (Beyotime, Shanghai, China). Then the proteins were subjected to sodium dodecyl sulfate-polyacrylamide gel electrophoresis, using 10% SurePAGE-gels (Cat Number: M00665, GenScript, Piscataway, NJ, USA). Proteins were transferred to polyvinylidene difluoride (PVDF) membranes using a Trans-Blot Mini Cell (Bio-Rad). For immunodetection, the blots were blocked with 2% BSA in TBST (TBS+ 0.1% Tween-20) for 1 h at room temperature (RT) and kept with primary antibodies PSD-95 (1:1000, Cat Number: 51-6900, Invitrogen, Camarillo, CA, USA) or GluA1 (1:1000, Cat Number: ab31232, Abcam, Cambridge, MA, USA) or GAPDH (1:10,000, Cat Number: AC001, ABclonal, Woburn, MA, USA) at 4 °C overnight. The next day, the blots were washed three times in TBST and incubated with goat anti-rabbit IgG(H+L) (1:10,000, Cat Number: AS070, ABclonal, Woburn, MA, USA) for 1 h at room temperature. After the last three times washes, the bands were detected by Western Blotting Detection System (GE Healthcare Bioscience), and images were captured using a ChemiDoc™ Touch Imaging System (Bio-Rad Laboratories, Hercules, CA). The images were analyzed by the Image LabTM 3.0 software (Bio-Rad Laboratories).

### Measurement of tumor necrosis factor α (TNF-α) and interleukin-6 (IL-6)

ELISA kits were used for the measurement of plasma levels of TNF-α (Cat Number: 88-7324-88, Invitrogen, Carlsbad, CA, USA) and IL-6 (Cat Number: 88-7064-88, Invitrogen, Carlsbad, CA, USA) according to the manufacturer’s instructions.

### 16S rRNA analysis

The DNA extraction from fecal samples and the 16S rRNA analysis was performed at Majorbio Bio-Pharm Technology Co. Ltd. (Shanghai, China). The analysis of 16S rRNA from fecal samples was performed as previously described. Briefly, PCR was performed using 338 F (5'-ACTCCTACGGGAGGCAGCAG-3') and 806 R (5'-GGACTACHVGGGTWTCTAAT-3') by thermocycler PCR system (GeneAmp 9700, ABI, USA) to amplify the V3–V4 region of the bacterial 16S rRNA gene. The amplified DNA (~330 bp) was purified using the AxyPrep DNA Gel Extraction Kit (Axygen Biosciences, Union City, CA, USA) and quantified using QuantiFluor ™ -ST (Promega, USA). The 16S amplicons were then sequenced using MiSeq platform according to the Illumina protocol. Raw fastq files were quality-filtered by Trimmomatic and merged by FLASH with the following criteria: (i) The reads were truncated at any site receiving an average quality score <20 over a 50 bp sliding window. (ii) Sequences whose overlap being >10 bp were merged according to their overlap with mismatch no >2 bp. (iii) Sequences of each sample were separated according to barcodes (exactly matching) and Primers (allowing 2 nucleotide mismatching), and reads containing ambiguous bases were removed. Operational taxonomic units (OTUs) were clustered with 97% similarity cutoff using UPARSE (version 7.1 http://drive5.com/uparse/) with a novel ‘greedy’ algorithm that performs chimera filtering and OTU clustering simultaneously. The taxonomy of each 16S rRNA gene sequence was analyzed by RDP Classifier algorithm (http://rdp.cme.msu.edu/) against the Silva (SSU123) 16S rRNA database using a confidence threshold of 70%.

The alpha diversity was used to analyze the complexity of species diversity for each sample using four indices: Chao1, ACE, and Shannon. The beta diversity of unweighted UniFrac distances was analyzed. Differences in the bacterial taxa between groups at the species level or higher (depending on the taxon annotation) were calculated using linear discriminant analysis (LDA) effect size (LEfSe) with LEfSe software [33].

### Flow cytometry

Mouse spleen and Peyer’s patches (PPs) were mashed and passed through 70 μm mesh to prepare a single-cell suspension for FACS analysis. Single-cell suspensions from the spleen were processed by red blood cell lysate buffer and then used for staining. For regulatory T cell quantification, the following antibodies were used: anti CD3e, APC-Cy7 (Cat Number: 557596, BD Bioscience), anti CD4, PerCP-Cy5.5 (Cat Number: 550954, BD Bioscience), anti CD25, FITC (Cat Number: 102006, BioLegend), anti FOXP3, APC (Cat Number: 17-5773-82, eBioscience). Around 1 × 10^6^ cells were seeded into a 96-well plate and stimulated by phorbol myristate acetate (50 ng/ml), ionomycin (1 μg/ml) and golgi inhibitor (1:1000, BD GolgiPlug, Cat Number: 51-2301kz) for Th1/Th2/Th17 cell subset analysis. Then cells were harvested and stained with the following antibodies: anti INF-γ, FITC (Cat Number: 505806, BioLegend), anti-IL-17A, PE (Cat Number: 506904, BioLegend), anti-IL-4, APC (Cat Number: 504105, BioLegend) and anti CD3e, CD4 antibodies as described above. Cells were detected using a BD FACSCalibur flow cytometer (BD Biosciences) and were analyzed using FlowJo software (Version 7.6.1).

### Statistical analysis

The animal experiment data were presented as the mean ± standard error of the mean (SEM). The statistical analysis was performed using SPSS Statistics 20 (SPSS, Tokyo, Japan). A test of homogeneity of variance for all animal data showed no significant difference. Data of body weight were analyzed using repeated two-way analysis of variance (ANOVA), followed by *post-hoc* Tukey’s multiple comparison test. Data except gut microbiota were analyzed by Student *t*-test. Data of 16S rRNA were analyzed by Mann–Whitney *U*-test. Correlation was determined by Pearson correlation. The *P*-values of <0.05 were considered statistically significant.

## Results

### Depressive-like behaviors, systemic inflammation and decreased expression of synaptic proteins in the PFC of CIA mice

The body weight of CIA mice was significantly decreased since the first injection compared to control mice (Fig. 1B). The joints of CIA mice have turned to red and swollen from day 14 and insistently aggravated like the clinical score, and the H&E staining showed abnormalities in the CIA mice (Fig. 1C, D). For behavioral tests, there were no changes in locomotion between the two groups (Fig. 1E). The immobility time of the TST and FST in the CIA mice were significantly higher than those of the control mice (Fig. 1F, G). In the SPT, the CIA mice exhibited significantly decreased sucrose preference compared to control mice (Fig. 1H). The plasma levels of IL-6 in the CIA group were significantly higher than control group although plasma levels of TNF-α were not altered in the two groups (Fig. 1I, J). Western blot analysis showed the decreased expression of synaptic proteins (e.g., PSD-95 and GluA1) in the PFC from CIA mice compared to control mice (Fig. 1K, L).

These data suggest that CIA mice show depression-like phenotypes, systemic inflammation, and decreased expression of synaptic proteins in the PFC.

### Abnormal composition of gut microbiota after CIA

We performed 16S ribosome RNA sequencing analysis of the fecal samples of the two groups. For α-diversity, ACE index in the CIA group was significantly decreased compared to the control group, although other indices of α-diversity (Chao1, Shannon, ACE) were not altered in the two groups (Fig. S1A). The unweighted UniFrac distance between the two groups showed no changes (*R* = −0.0237, *P* = 0.569) for β-diversity (Fig. S1B).

At the phylum level, the abundance of *Actinobacteriota* in the CIA mice was significantly higher than that in the control mice (Fig. S2A). At the genus level, the abundance of *Enterorhabdus* in the CIA group was significantly higher than that in the control (Fig. S2B). At the species level, *Alistipes sp. cv1* significantly decreased in the CIA group compared with control group (Fig. S3A, B). In contrast, *Bacteroides vulgatus, Lachnospiraceae bacterium COE1, Lachnospiraceae bacterium 28-4* were significantly increased in the CIA group compared with the control group (Fig. S3C–E).

Next, we used the LEfSe algorithm to identify the microbial markers that are more important in one group than in the other group (Fig. S3F). We identified the different distributions of three bacteria (e.g., *Eggerthellaceae, Coriobacteriales, Coriobacteriia*) for the CIA mice (Fig. S3F). Seven mixed-level phylotypes, including *Enterorhabdus, Eggerthellaceae, Coriobacteriia, Coriobacteriales, Actinobacteriota, Lachnospiraceae bacterium 28-4, Eubacterium xylanophilum group* were identified as potential microbial markers for the CIA group (Fig. S3G).

### Effects of FMT from RA patients to ABX-treated mice

Next, we investigated whether FMT from RA patients or HCs to ABX-treated mice could produce depression-like phenotypes (Fig. 2A). There was no difference in locomotor activity between the two groups (Fig. 2B). In contrast, FMT from RA patients significantly increased the immobility time of the TST and the FST in the ABX-treated mice compared to FMT from HCs (Fig. 2C, D). Furthermore, the sucrose preference in the SPT after FMT from RA patients was significantly lower than those of FMT from HCs (Fig. 2E). Moreover, plasma levels of TNF-α and IL-6 in the group of FMT from RA patients were significantly higher than those of FMT from HCs (Fig. 2F, G). Western blot analysis showed the decreased expression of synaptic proteins (e.g., PSD-95 and GluA1) in the PFC from ABX-treated mice after FMT from RA patients compared to FMT from HCs (Fig. 2H, I).

These data suggest that FMT from RA patients could cause depression-like phenotypes, systemic inflammation, and decreased expression of synaptic proteins in the PFC.

### Altered gut microbiota composition in the ABX-treated mice after FMT from RA patients

**Fig. 3:**
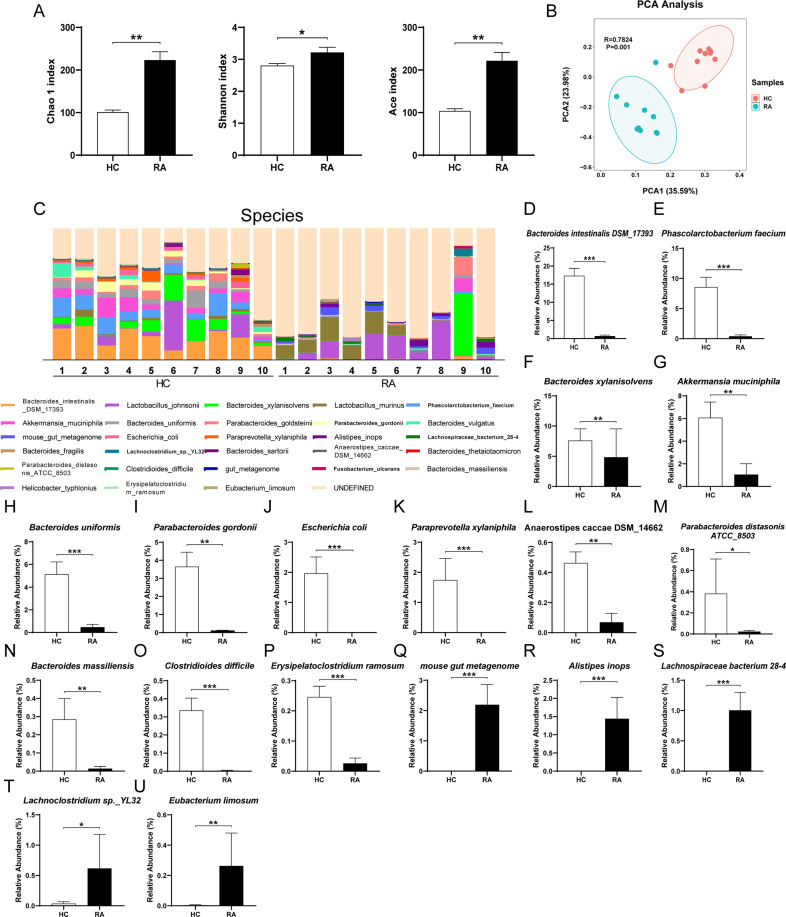
Altered gut bacteria composition at the species level after FMT. **A** Alpha diversity indices (i.e., Chao1, Shannon, ACE). Chao1 (Mann–Whitney *U*-test: *U* = 10, *P* = 0.002). Shannon (Mann–Whitney *U*-test: *U* = 17, *P* = 0.013). ACE (Mann–Whitney *U*-test: *U* = 10, *P* = 0.002). **B** Principal component analysis (PCA) of beta-diversity based on the OTU table, where each point represents a single sample colored by group, indicated by the second principal component of 23.98% on the *Y-*axis and the first principal component of 35.59% on the *X*-axis (ANOSIM) (*R* = 0.7824, *P* = 0.001). **C** Relative abundance at the species level in the FMT group from RA patients and the FMT group from healthy controls. **D**–**U** Significantly different bacteria in species level from two groups. **D** *Bacteroides intestinalis DSM_17393*. **E** *Phascolarctobacterium faecium*. **F** *Bacteroides xylanisolvens*. **G** *Akkermansia muciniphila*. **H** *Bacteroides uniformis*. **I** *Parabacteroides goldsteinii*. **J** *Escherichia coli*. **K** *Paraprevotella xylaniphila*. **L** *Anaerostipes caccae DSM_14662*. **M** *Parabacteroides distasonis ATCC_8503*. **N** *Bacteroides massiliensis*. **O** *Clostridioides difficile*. **P** *Erysipelatoclostridium ramosum*. **Q** *mouse gut metagenome*. **R** *Alistipes inops*. **S** *Lachnospiraceae bacterium 28-4*. **T** *Lachnoclostridium sp._YL32*. **U** *Eubacterium limosum*. See Table S3 for detailed statistical analysis. Data are shown as mean ± SEM (*n* = 10). *P < 0.05, **P < 0.01, ***P < 0.001.

We analyzed α-diversity and β-diversity of mice after FMT from RA patients or HCs. All three kinds of α-diversity (Chao1, Shannon, ACE) were significantly increased in the group of FMT from RA patients compared with FMT from HCs (Fig. 3A). Principal component analysis (PCA) showed a remarkable difference in microbiota composition between the two groups (Fig. 3B).

Furthermore, the relative abundance was used to define differential bacteria between the two groups at three different levels. Five significantly different phyla were clarified (e.g., *Verrucomicrobiota, Proteobacteria, Actinobacteriota*) (Fig. S4A-F). At the genus level, the abundance of 27 genera (*Bacteroides, Lachnoclostridium, Phascolarctobacterium*, etc.) were significantly different between the two groups (Fig. S5AB and Table S2). At the species level, eighteen species were found significantly changed between the two groups (Fig. 3C–U and Table S3). The most abundant bacteria in FMT from the HCs (e.g., *Bacteroides intestinalis DSM_17393, Phascolarctobacterium faecium, Bacteroides xylanisolvens, Akkermansia muciniphila*) were significantly decreased compared to FMT from the RA patients (Fig. 3D-G). In contrast, species including *mouse gut metagenome, Alistipes inops, Lachnospiraceae bacterium 28-4, Lachnoclostridium sp._YL32, Eubacterium limosum* were significantly increased after FMT from RA patients compared with FMT from HCs (Fig. 3Q–U).

### Effects of FMT from RA patients on the LEfSe algorithm of gut microbiota

We used LEfSe algorithm analysis for high-dimensional biomarker discovery among the two groups by the OUT data. Different colors represent different abundant taxon and different microbial biomarkers. The classification of potential microbial markers was presented in the two groups (Fig. S6A). Twelve mixed-level phylotypes, including *Muribaculaceae, Alistipes inops, Alistipes, Rikenellaceae, Blautia, Actinobacteriota, Lachnospiraceae bacterium 28-4, Coriobacteriales, Coriobacteriia, Eggerthellaceae, Faecalibaculum,* and *Ruminococcus torques group* were identified as potential microbial markers in the FMT group from RA patients compared with the FMT group from HCs (Fig. S6B).

### Effects of FMT from RA patients in FACS analysis and correlation analysis

**Fig. 4:**
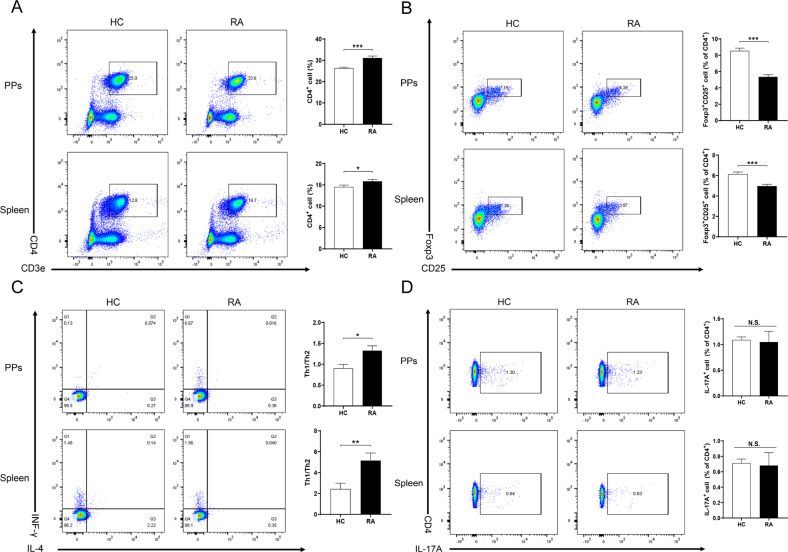
FACS analysis of PPs and spleen samples between two groups after FMT from RA patients or healthy controls. **A** Representative images of different expression of CD4^+^ T cells (% of total cells) in PPs and spleen. PPs (Student *t*-test: *t* = −4.944, *P* < 0.001); spleen (Student *t*-test: *t* = −2.258, *P* = 0.036). **B** Representative images of different expression of Treg cells (Foxp3^+^CD25^+^, % of CD4^+^ T cell) in PPs and spleen. PPs (Student *t*-test: *t* = 7.443, *P* < 0.001); spleen (Student *t*-test: *t* = 4.290, *P* < 0.001). **C** Representative images of different expression of Th1 cells (IFN-γ^+^, % of CD4^+^ T cell) / Th2 cells (IL-4^+^, % of CD4^+^ T cell) in PPs and spleen. PPs (Student *t*-test: *t* = −2.860, *P* = 0.010); spleen (Student *t*-test: *t* = −2.955, *P* = 0.008). **D** Representative images of different expression of Th17 cells (IL-17A^+^, % of CD4^+^ T cell). PPs (Student *t*-test: *t* = −0.042, *P* = 0.967); spleen (Student *t*-test: *t* = 0.426, *P* = 0.675).

In addition to Peyer’s patches, the spleen, the largest immune organ in the body, is also closely related to depression [34–36]. Therefore, we used the single-cell suspension from PPs and spleen for FACS analysis. Compared to FMT from HCs, FMT from RA patients increased the percentage of T-lymphocyte (CD3e^+^ CD4^+^) in PPs and spleen, indicating a set of immune activation (Fig. 4A). Furthermore, the percentage of Treg (CD25^+^ Foxp3^+^) cells was notably decreased after FMT from RA patients compared with FMT from HCs (Fig. 4B). In contrast, the index of Th1/Th2 (INF-γ^+^IL-4^-^ cells/INF-γ^-^IL-4^+^ cells) significantly increased in the FMT group from RA patients than the FMT group from HCs (Fig. 4C). There was no difference in Th17 (CD4^+^IL-17^+^) cells between the two groups (Fig. 4D).

**Fig. 5:**
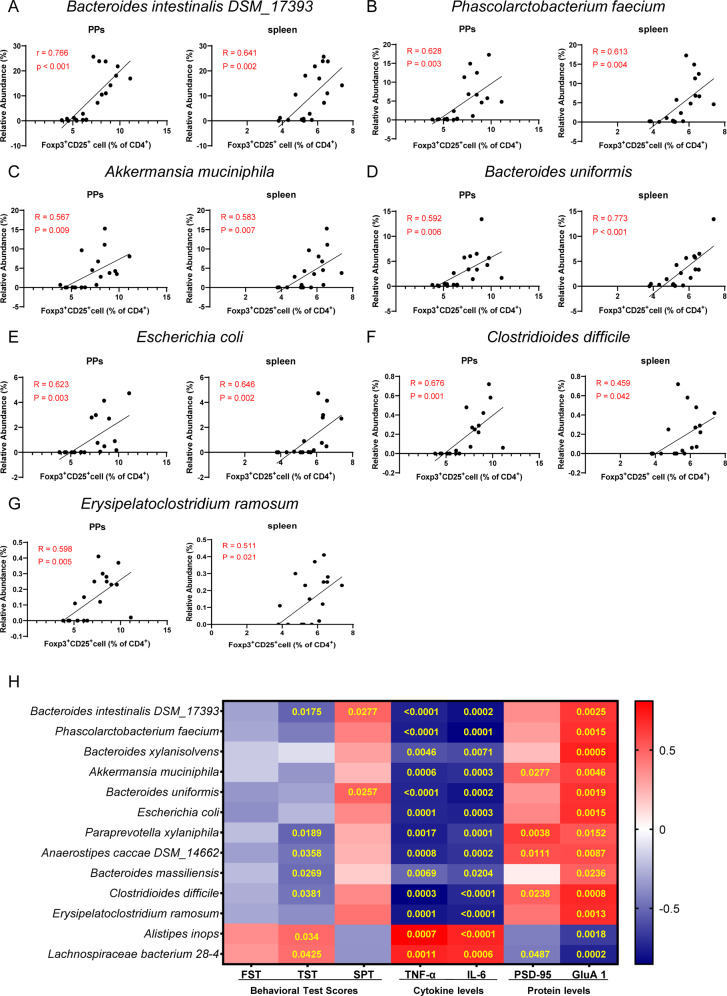
Correlation of bacteria-species with Treg cell from PPs and spleen. **A** *Bacteroides intestinalis DSM_17393*. **B** *Phascolarctobacterium_faecium*. **C** *Akkermansia muciniphila*. **D** *Bacteroides uniformis*. **E** *Escherichia coli*. **F** *Clostridioides difficile*. **G** *Erysipelatoclostridium ramosum*. **H** The heat map displayed the correlation coefficient between bacterial abundance and the results of behavioral tests, cytokines, and western blot in the PFC. The yellow text in the center of each box represents the *P*-value.

In the correlation analysis, the relative abundance of *Bacteroides intestinalis DSM_17393, Phascolarctobacterium faecium, Akkermansia muciniphila, Bacteroides uniformis, Escherichia coli, Clostridioides difficile, Erysipelatoclostridium ramosum* were positively correlated with the level of Treg cells both in PPs and spleen (Fig. 5A–G). Similar to the data of Treg cells, the relative abundance of several bacteria (e.g., *Bacteroides intestinalis DSM_17393, Phascolarctobacterium faecium, Escherichia coli*) exhibited a negative correlation between Th1/Th2 index (Fig. S7A–C).

Finally, we used the heat maps to show the correlations between the relative abundance of the species microbiota and the results of behavioral tests, pro-inflammatory cytokines, and expression of synaptic proteins (PSD-95 and GluA1) in the PFC in FMT from RA patients (Fig. 5H). There were correlations between relative abundance of microbiome and depression-like phenotypes (Fig. 5H). Furthermore, plasma levels of pro-inflammatory cytokines (TNF-α, IL-6) and the synaptic proteins in the PFC were correlated with alterations of species microbiome (Fig. 5H).

These findings suggest that abnormal composition of gut microbiota may affect systemic inflammation and synaptic proteins in the PFC via gut - microbiota - brain axis, resulting in depression-like phenotypes.

## Discussion

The major findings of this study were as follows. First, in addition to RA-like symptoms, CIA mice exhibited depression-like behaviors, elevated blood levels of IL-6, downregulation of synaptic proteins (e.g., PSD-95 and GluA1) in the PFC, and abnormal composition of gut microbiota. Second, FMT from RA patients caused depression-like behaviors in the ABX-treated mice through systemic inflammation. Analysis using PCA showed that the gut microbiota was completely different between the two FMT groups. Further analysis by LEfSe algorithm showed twelve possible biomarkers for the FMT group from RA patients. There were correlations between the relative abundance of microbiota and behavioral data, plasma cytokines, or synaptic proteins in the PFC. Third, CD4^+^ T cells and the ratio of Th1/Th2 in the PPs and spleen were increased in the FMT group from RA patients compared to FMT from HCs, and the proportion of Treg cells decreased in the FMT group from RA patients. Fourth, there were positive correlations between three species bacteria and T cells both in PPs and spleen. Finally, there were significant correlations between the relative abundance of microbiome and behavioral outcome, blood cytokines or synaptic protein in the PFC. Collectively, the present data indicate that FMT from RA patients could activate abnormal differentiation of T cells by changing the gut microbiota in the ABX-treated mice, resulting in depression-like phenotypes.

Depression is one of the most important complications of RA patients [37, 38]. Depression is also associated with poor treatment response and the increased disability and mortality of RA patients [5, 39–42]. A meta-analysis involving 70,000 RA patients shows that the probability of RA patients suffering from MDD is 18% and depression is 38.8%, far above the percentage in the general population [43]. In this study, we found that CIA mice showed depression-like behaviors, consistent with the previous report that sucrose preference decreased in CIA mice [44]. These results show that CIA mice exhibit depression-like phenotypes, systemic inflammation, and abnormal composition of gut microbiota, in agreement with clinical experience in RA patients.

Treatment with ABX is known to induce dramatic alterations in the diversity and composition of the gut microbiota in the host intestine [45, 46]. We previously reported that FMT from *Chrna7* KO mice with depression-like phenotypes caused depression-like behaviors, higher blood levels of IL-6, and downregulation of synaptic proteins in the PFC in ABX-treated mice [20]. In contrast, FMT from mice with depression-like phenotype did not cause depression-like phenotypes in water (no ABX)-treated mice [22]. Thus, it is likely that ABX-induced microbiome depletion is required for these behavioral and biochemical changes in recipient mice after oral injection of “depression-related microbes” obtained from mice with depression-like phenotypes [22–24]. In this study, we found that FMT from RA patients caused depression-like behaviors, systemic inflammation, T cell activation, and reduced synaptic proteins in ABX-treated mice. It seems that feces from RA patients might include “depression-related microbes”. Although the precise mechanisms underlying the depression-like phenotypes in ABX-treated mice caused by FMT of “depression-related microbes” from RA patients remain unknown, it seems that gut–microbiota–brain axis through abnormal T cells differentiation in the recipient mice plays a role in the depression-like phenotypes. Further study is needed to ascertain the role of T cells differentiation in gut–microbiota–brain axis.

It is suggested that Tregs play a protective role against depression-like behaviors in rodents. A previous study showed that the proportion of Tregs was negatively correlated with decreased sucrose preference in the chronic unpredictable mild stress model of depression [47]. Importantly, Tregs exhaustion before mice exposed to stress led to a higher rate of depressive-like behavior and pro-inflammatory profile than controls [48]. It is noteworthy that FMT from RA patients decreased the percentage of Treg cells compared with FMT from HCs. Increases in Th1/Th2, as an indicator of inflammation, can trigger an inflammatory response in several diseases [49]. In this study, we found an increase in the ratio of Th1/Th2 in mice after FMT from RA patients. Taken together, it is likely that alterations in the gut microbiota might cause the decrease of Treg cells and the increase of Th1/Th2 index, which leads to systemic inflammation, resulting in depression-like behaviors.

Interestingly, we found three kinds of species (e.g., *B. intestinalis DSM_17393, P. faecium and E. coli*), which showed a positive correlation with Treg cells and a negative correlation with the ratio of Th1/Th2. It seems that the decrease of these three kinds of the species stimulated the differentiation of T cells to Th1 cells rather than Tregs and Th2 cells. Furthermore, we found that these three species were negatively correlated with the inflammatory cytokines TNF-α and IL-6, and were positively correlated with synaptic protein in the PFC. We found correlations between the relative abundance of *B. intestinalis DSM_17393* and depression-like behaviors, suggesting that *B. intestinalis DSM_17393* may be associated with depression-like behaviors in mice after FMT from RA patients. The three species microbiome are the main abundant bacteria in healthy controls. *Bacteroides*, the genus of *B. intestinalis DSM_17393*, decreased in chronic social defeat stress model of depression [50]. *P. faecium* abundant colonization in the human gastrointestinal tract, are more susceptible to digestive and metabolic diseases [51]. Oral gavage of *E. coli* caused colitis, cognitive decline, and depression-like behavior in mice [52]. In the Fig. 5H, there were negative correlations between a number of microbiome and plasma cytokines. Although the detailed functions of these microbiome remain unclear, it is possible that these microbes may trigger inflammatory response in the host although further study is needed.

In this study, we found that α-diversity and β-diversity of gut microbiota were no changes between control mice and CIA mice, whereas the diversity in mice after FMT from RA patients was altered compared to control. One possibility is the vast difference between the bacteria in human feces and mouse feces, suggesting that FMT from human bacteria dramatically could alter the gut microbiota in mice. Interestingly, the phyla *Actinobacteriota*, the genus *Enterorhabdus* and the species *L. bacterium 28-4* exhibited significant changes in both models, suggesting that these three bacterial taxa may be associated with depression-like phenotypes. In addition, we found that the relative abundance of *A. inops* and *L. bacterium 28-4* were positively correlated with inflammatory cytokines (Fig. 5H). A recent review suggests that the genus *Alistipes* including *A. inops* is associated with inflammation, cancer, and mental health [53]. It is likely that the increase of these two bacteria may play a role in the systemic inflammation, resulting in depression-like behaviors via gut–microbiota–brain axis.

In this study, there were no changes in blood levels of TNF-α between control mice and CIA mice. However, FMT from RA patients caused significant increase in blood levels of TNF-α in ABX-treated mice. Although the reasons underlying the discrepancy is currently unknown, it is possible that FMT from RA patients may produce systemic inflammation through gut microbiota with inflammatory actions.

This study has some limitations. First, we did not measure the severity of depression in the RA patients in this study. Further study of FMT from RA patients with depressive scores (mild to severe) is important. It is, therefore, interesting to study whether clinical severity of RA patients with depression could contribute depression-like behaviors in ABX-treated mice. Second, RA patients are frequently afflicted by pain which may affect depression [54]. However, we did not measure pain scores in CIA mice and mice after FMT from RA patients. It is also of interest to examine whether pain might affect depression-like behaviors in CIA mice or ABX-treated mice after FMT from RA patients. Third, the recent studies demonstrated the role of subdiaphragmatic vagus nerve on the depression-like behaviors in ABX-treated mice after FMT of depression-related microbes [20, 22, 24, 55, 56]. Further study on the role of subdiaphragmatic vagus nerve on depression-like behaviors in rodents after FMT from RA patients is needed.

In conclusion, this study showed that CIA mice show depression-like behaviors, systemic inflammation, and abnormal composition of gut microbiota, and that FMT of “depression-related microbes” from RA patients caused depression-like phenotypes in ABX-treated mice through the activation of T cells immunization. Further studies of the role of the gut–microbiota–brain axis via the T cells in RA and depression are needed.

## Supplementary information

Supplemental information

## Data Availability

The 16S rRNA sequencing data have been deposited to the NCBI Sequence Read Archive and are available at the accession number PRJNA783164.
