# Correction: Fecal microbiota transplantation from patients with rheumatoid arthritis causes depression-like behaviors in mice through abnormal T cells activation

**DOI:** 10.1038/s41398-022-02168-6

**Published:** 2022-09-16

**Authors:** Yaoyu Pu, Qiuping Zhang, Zhigang Tang, Chenyang Lu, Liang Wu, Yutong Zhong, Yuehong Chen, Kenji Hashimoto, Yubin Luo, Yi Liu

**Affiliations:** 1 Department of Rheumatology and Immunology, West China Hospital, Sichuan University, Chengdu, Sichuan 610041 China; 2 Division of Clinical Neuroscience, Chiba University Center for Forensic Mental Health, Chiba, 260-8670 Japan

**Keywords:** Depression, Diagnostic markers

Correction to: *Translational Psychiatry* 10.1038/s41398-022-01993-z, published online 01 June 2022

The original version of this article unfortunately contained a mistake. The authors state the following: After our article was published, we found the errors in the method section of Western blot analysis. These mistakes do not affect the results of this article. The authors apologize for the errors in the methods. The corrected version of method has been provided and replaced. The original article has been corrected.

**Published method for Western blot analysis:**

“Proteins were transferred to polyvinylidene difluoride (PVDF) membranes using a Trans-Blot Mini Cell (Bio-Rad). For immunodetection, the blots were blocked with 2% BSA in TBST (TBS+ 0.1% Tween-20) for 1 h at room temperature (RT) and kept with primary antibodies PSD-95 (1:1000, Cat Number: 51-6900, Invitrogen, Camarillo, CA, USA) or GluA1 (1:1000, Cat Number: ab31232, Abcam, Cambridge, MA, USA) at 4 °C overnight. The next day, the blots were washed three times in TBST and incubated with GAPDH (1:10,000, Cat Number: AC002, ABclonal, Woburn, MA, USA) for 1 h at room temperature.”

**Corrected method for Western blot analysis:**

“Proteins were transferred to polyvinylidene difluoride (PVDF) membranes using a Trans-Blot Mini Cell (Bio-Rad). For immunodetection, the blots were blocked with 2% BSA in TBST (TBS+ 0.1% Tween-20) for 1 h at room temperature (RT) and kept with primary antibodies PSD-95 (1:1000, Cat Number: 51-6900, Invitrogen, Camarillo, CA, USA) or GluA1 (1:1000, Cat Number: ab31232, Abcam, Cambridge, MA, USA) or GAPDH (1:10,000, Cat Number: AC001, ABclonal, Woburn, MA, USA) at 4 °C overnight. The next day, the blots were washed three times in TBST and incubated with goat anti-rabbit IgG(H+L) (1:10,000, Cat Number: AS070, ABclonal, Woburn, MA, USA) for 1 h at room temperature.”

