# Supplementary material for: Fecal microbiota transplantation from patients with rheumatoid arthritis causes depression-like behaviors in mice through abnormal T cells activation
Source: Transl Psychiatry. 2022 Jun 1;12:223. doi: 10.1038/s41398-022-01993-z (PMC9160267; doi:10.1038/s41398-022-01993-z)
Supplement: Supplementary file 1 — Supplemental information [file 41398_2022_1993_MOESM1_ESM.docx]

**Supplemental information**

**Fecal microbiota transplantation from patients with rheumatoid arthritis cause depression-like behaviors in mice through abnormal T cells activation**

Yaoyu Pu^1^, Qiuping Zhang^1^, Zhigang Tang^1^, Chenyang Lu^1^, Liang Wu^1^, Yutong Zhong^1^, Yuehong Chen^1^, Kenji Hashimoto^2,*^, Yubin Luo^1,*^ and Yi Liu^1,*^

**Affiliations:** ^1^Department of Rheumatology and Immunology, West China Hospital, Sichuan University, Chengdu, Sichuan, China; ^2^Division of Clinical Neuroscience, Chiba University Center for Forensic Mental Health, Chiba 260-8670, Japan

*Corresponding authors

Supplemental figures: 7

Supplemental tables: 3

**
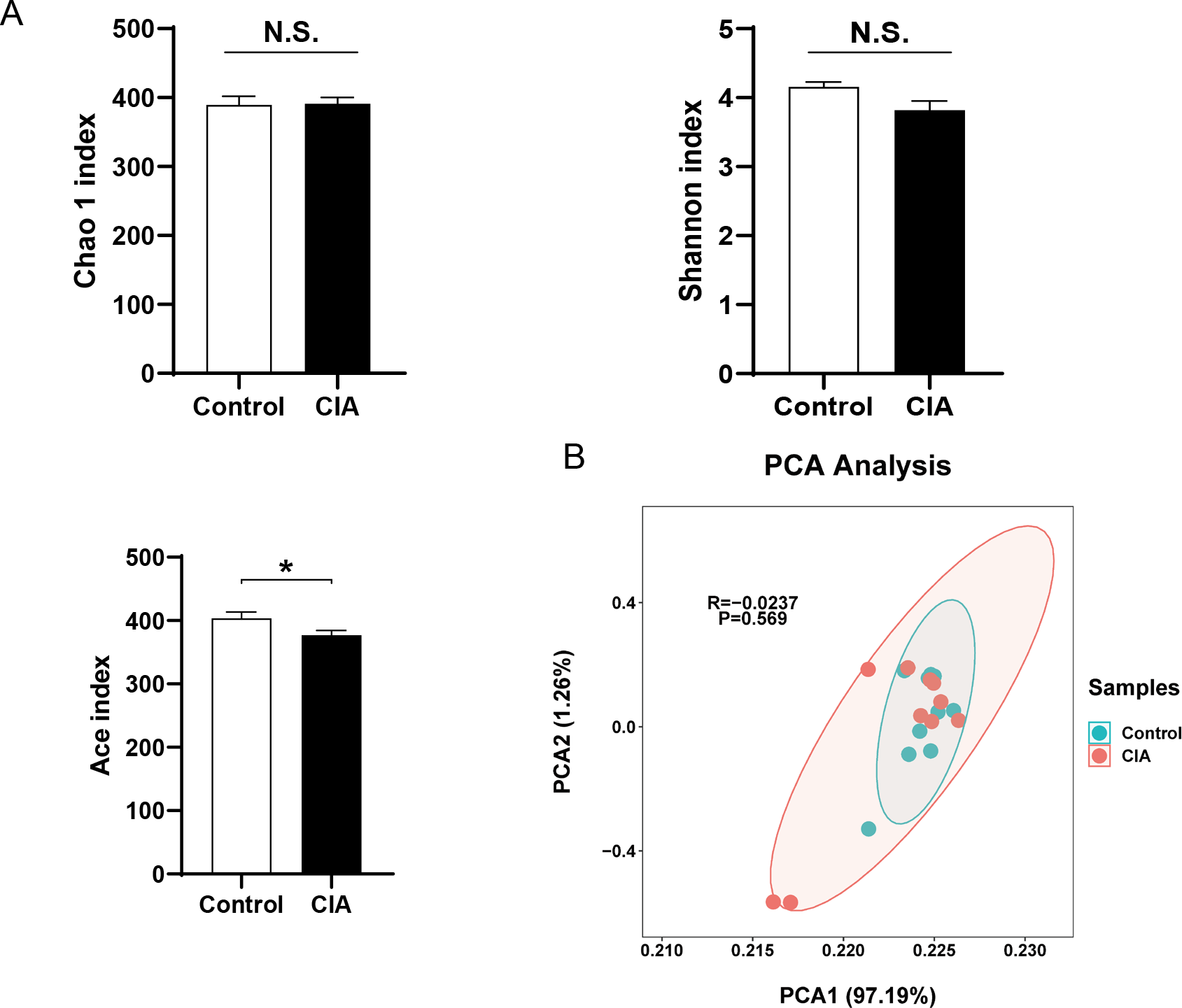
**

**Figure S1. α-diversity and β-diversity of gut microbiota in mice with collagen-induced arthritis (CIA)**

(A): Alpha diversity indices (i.e., Chao1, ACE, Shannon). Chao 1 (Mann-Whitney U-test: U = 45, P = 0.705). ACE (Mann-Whitney U-test: U = 12, P = 0.036) Shannon (Mann-Whitney U-test: U = 15, P = 0.074). (B): Principal component analysis (PCA) of beta-diversity based on the OTU table, where each point represents a single sample colored by group, indicated by the second principal component of 1.26% on the Y- axis and the first principal component of 97.19% on the X- axis (ANOSIM) (R = 0.0237, P = 0.569).


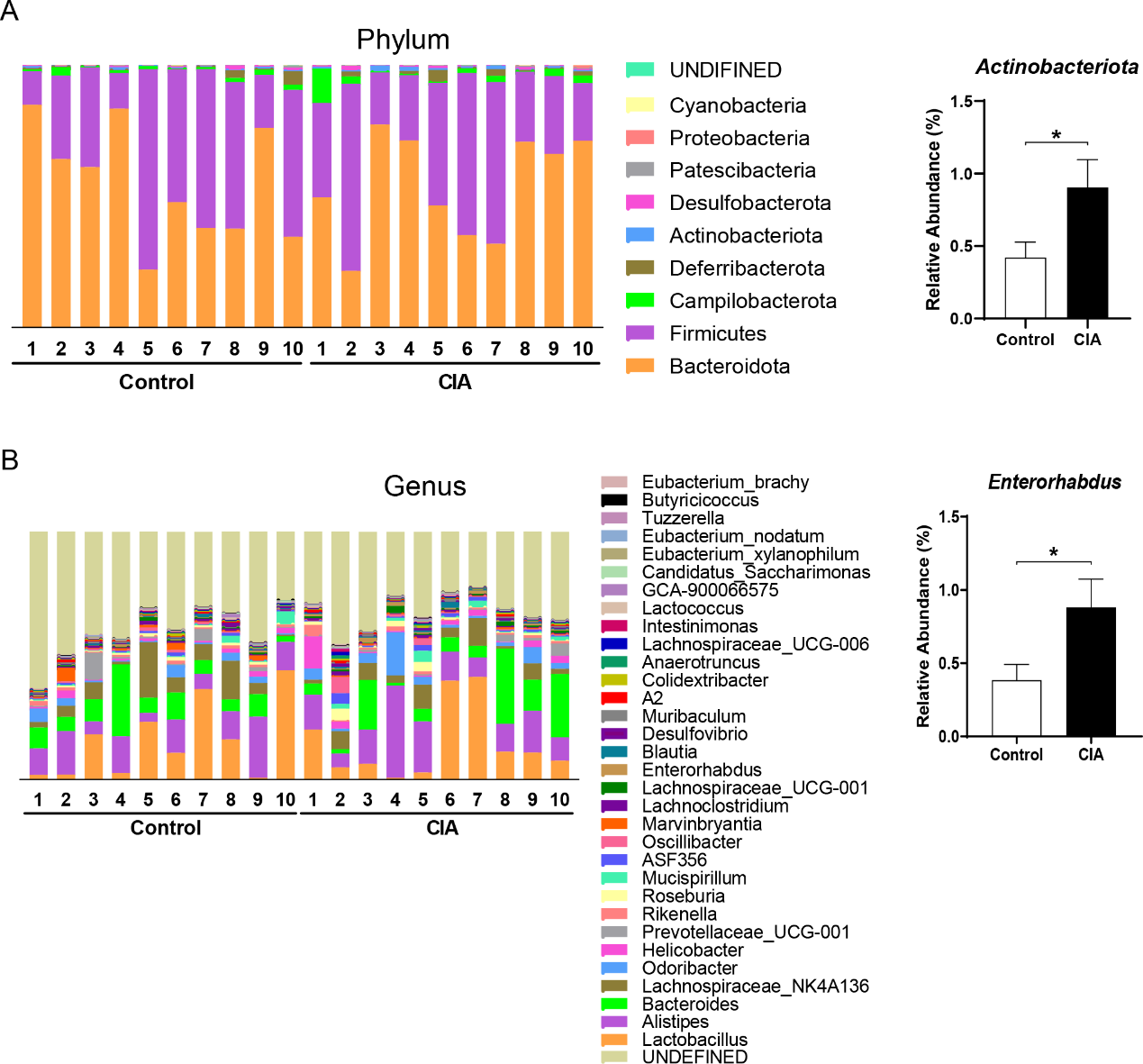


**Figure S2. Altered gut bacteria composition at the phylum and genus level after collagen-induced arthritis**

(A): Relative abundance at the phylum level. *Actinobacteriota* (Mann-Whitney U-test: U = 23, P = 0.041). (B): Relative abundance at the genus level. *Enterorhabdus* (Mann-Whitney U-test: U = 22.5, P = 0.038).

**
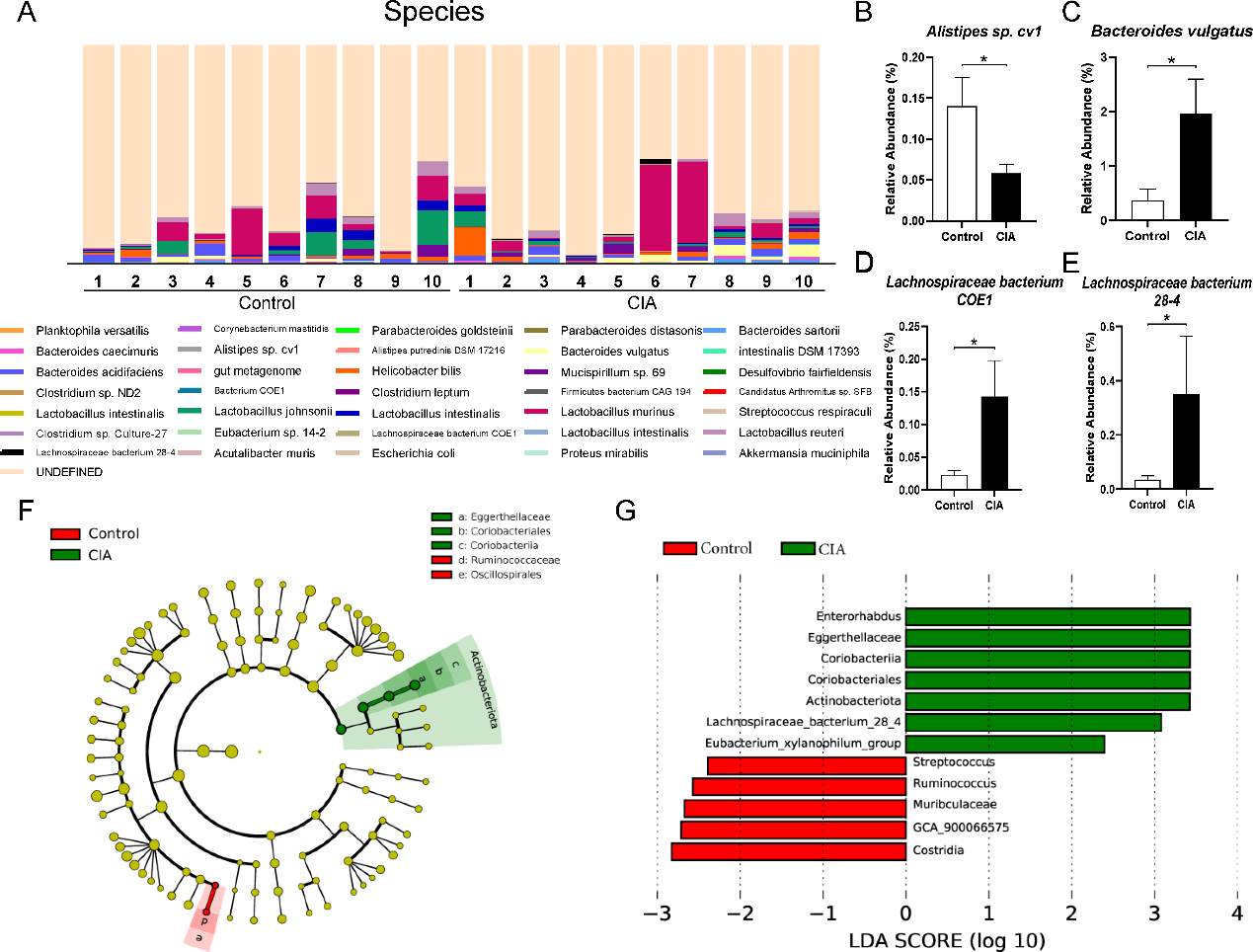
**

**Figure S3. Altered gut bacteria composition at the species level and LEfSe algorithm analysis**

(A): Relative abundance at the species level in the two groups. (B): *Alistipes sp. cv1* (Mann-Whitney U-test: U = 17, P = 0.037). (C): *Bacteroides vulgatus* (Mann-Whitney U-test: U = 18.5, P = 0.030). (D): *Lachnospiraceae bacterium COE1* (Mann-Whitney U-test: U = 17, P = 0.040). (E): *Lachnospiraceae bacterium 28-4* (Mann-Whitney U-test: U = 23.5, P = 0.044). (F): Cladogram (LDA score > 2.0, P < 0.05) showed the taxonomic distribution difference between the control and CIA mice, indicating the different color regions. Differential abundant taxonomic clades at phylum, class, order, family, genus and species level were showed by successive circles from the inner to outer rings. (G): Histograms of the different abundant taxa based on the cutoff value of LDA score (log10) > 2.0 and P < 0.05 between the two groups. The LDA scores of the control mice were negative, while those of CIA mice were positive. The data represent the mean ± SEM (n = 10). *P < 0.05.


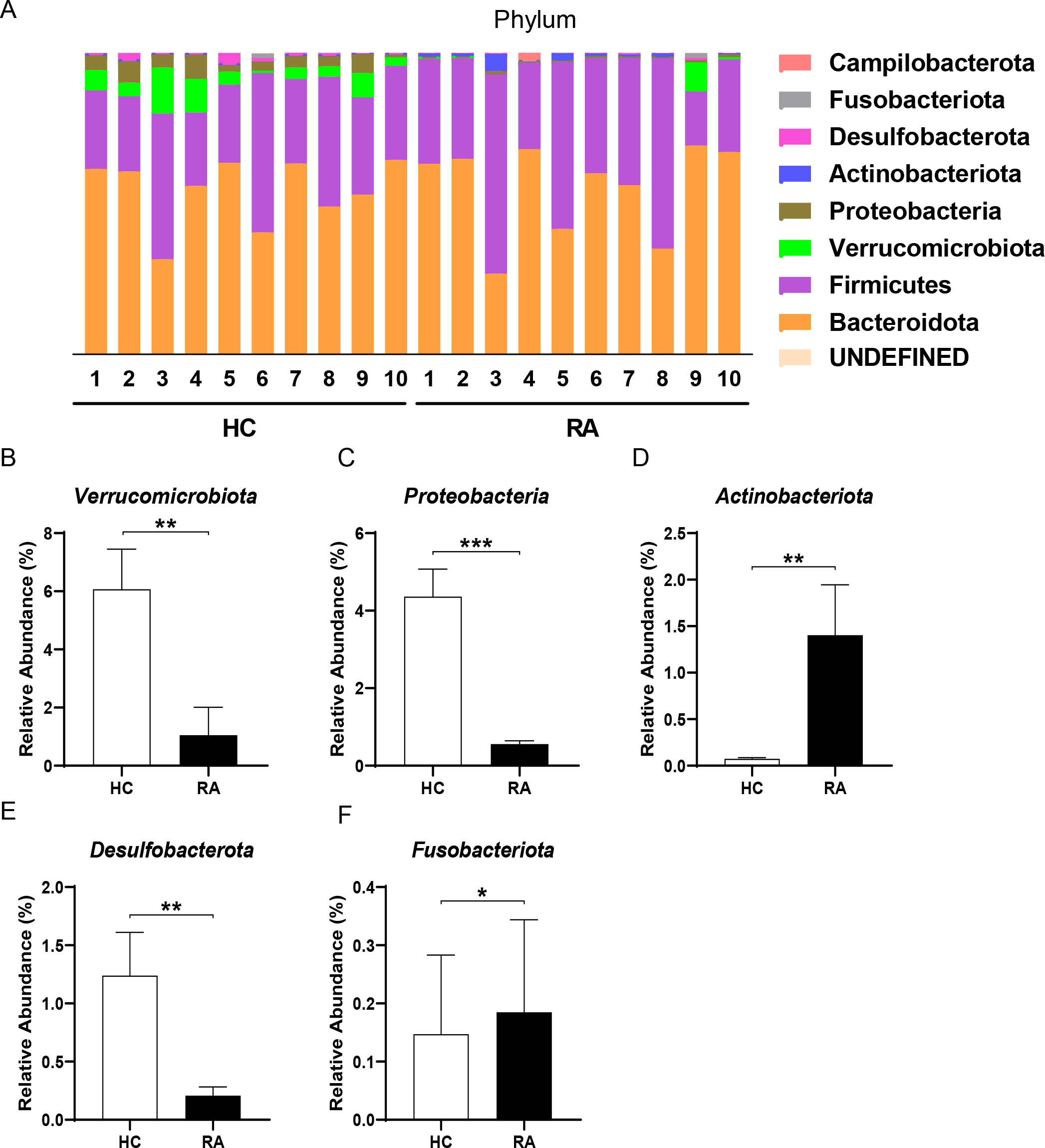


**Figure S4. Altered gut bacteria composition at the phylum level after FMT from RA patients or healthy controls**

(A): Relative abundance at the phylum level. (B): *Verrucomicrobiota* (Mann-Whitney U-test: U = 8, P = 0.001). (C): *Proteobacteria* (Mann-Whitney U-test: U = 2, P < 0.001). (D): *Actinobacteriota* (Mann-Whitney U-test: U = 11, P = 0.003). (E): *Desulfobacterota* (Mann-Whitney U-test: U = 8, P = 0.001). (F): *Fusobacteriota* (Mann-Whitney U-test: U = 24.5, P = 0.046).

**
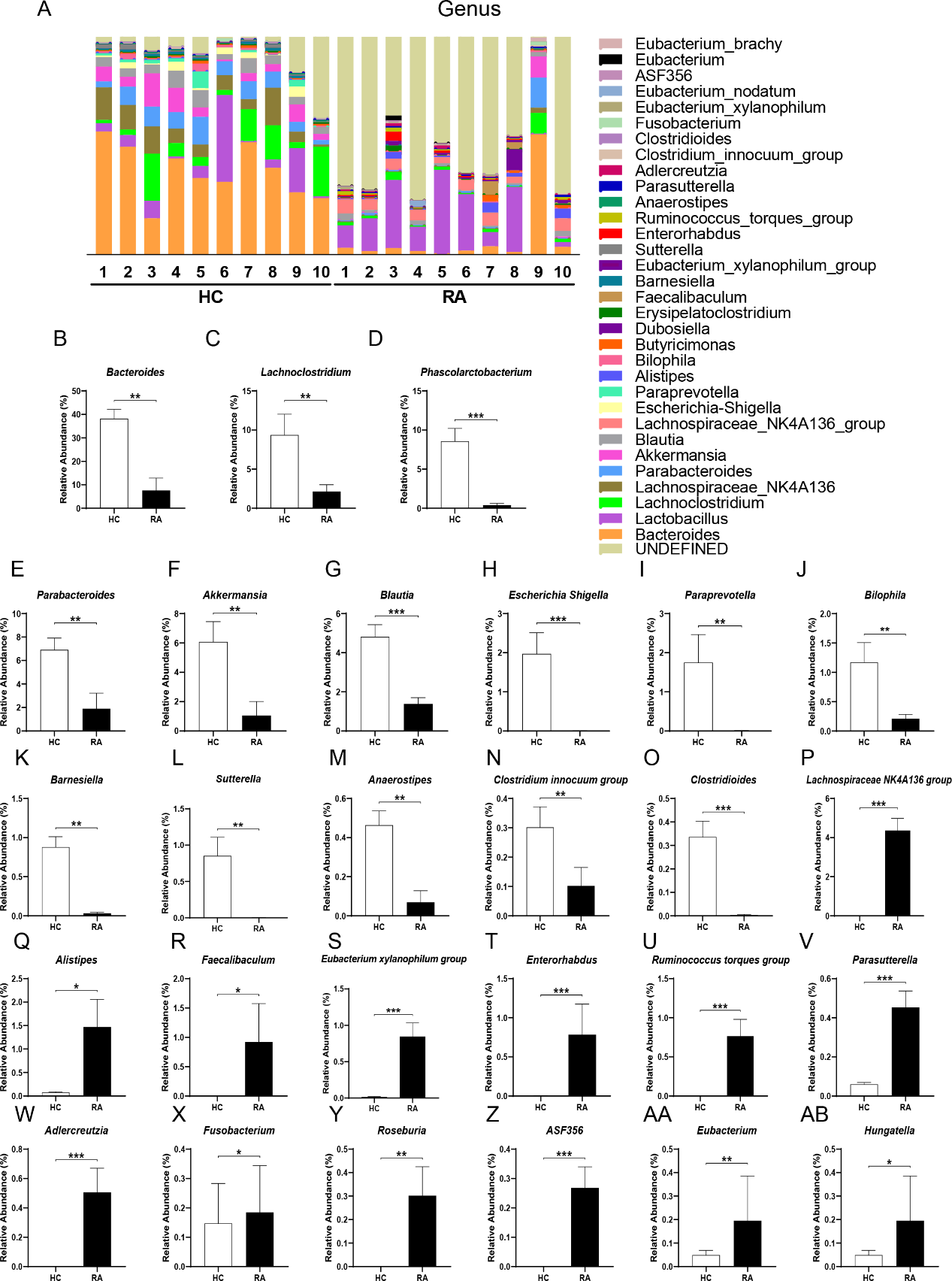
**

**Figure S5. Altered gut bacteria composition at the genus level after FMT.**

(A): Relative abundance at the genus level. (B): *Bacteroides*. (C): *Lachnoclostridium*. (D): *Phascolarctobacterium*. (E): *Parabacteroides*. (F): *Akkermansia*. (G): *Blautia*. (H): *Escherichia Shigella*. (I): *Paraprevotella*. (J): *Bilophila*. (K): *Barnesiella*. (L) *Sutterella*. (M): *Anaerostipes*. (N): *Clostridium innocuum group*. (O): *Clostridioides*. (P): *Lachnospiraceae NK4A136 group*. (Q): *Alistipes*. (R): *Faecalibaculum*. (S): *Eubacterium xylanophilum group*. (T): *Enterorhabdus*. (U): *Ruminococcus torques group*. (V): *Parasutterella*. (W): *Adlercreutzia*. (X): *Fusobacterium*. (Y): *Roseburia*. (Z): *ASF356*. (AA): *Eubacterium*. (AB): *Hungatella*.

See **Table S2** for detailed statistical analysis. Data are shown as mean ± S.E.M. (n = 10). *P < 0.05, **P < 0.01, ***P < 0.001.

**
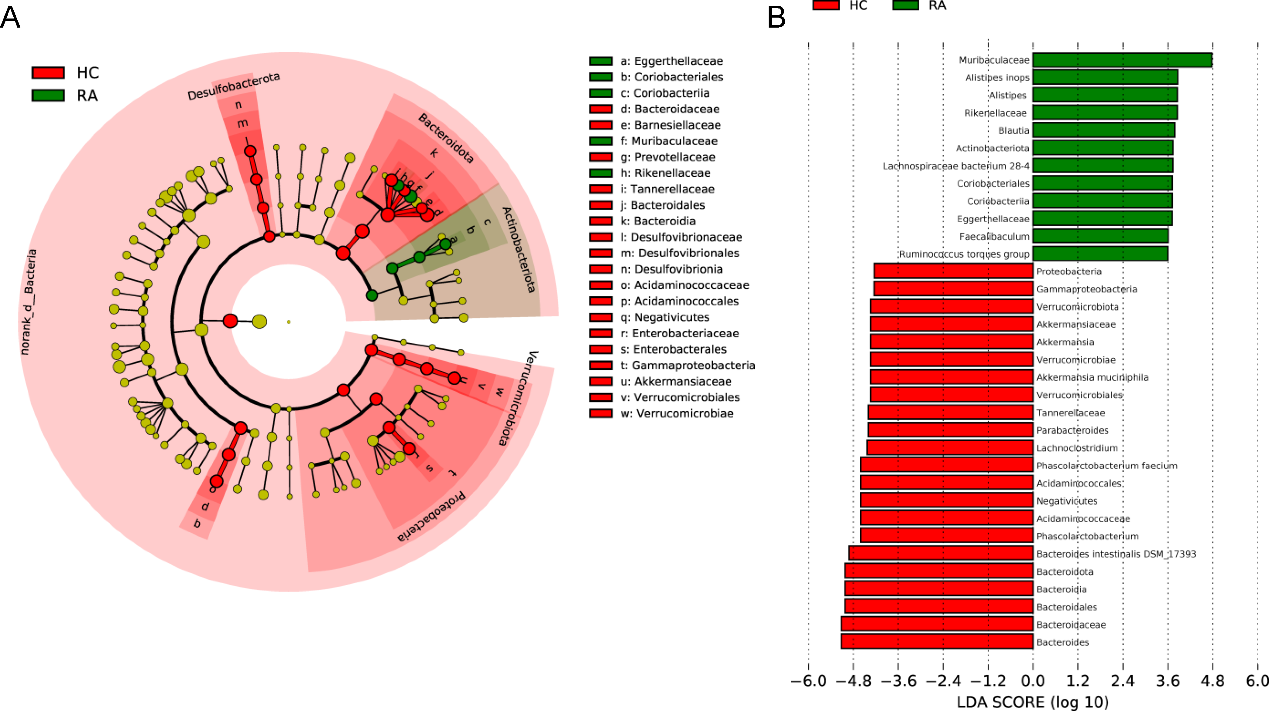
**

**Figure S6. Effects of FMT on LEfSe algorithm of gut microbiota**

Linear discriminant analysis Effect Size (LEfSe) algorithm of gut microbiota changes in abundant taxa between the two groups. The colors showed that the groups of abundant taxa were different from the other groups. (A): Cladogram (LDA score > 3.6, P < 0.05) showed the taxonomic distribution difference between the FMT group from RA patients and healthy controls, indicating by the different color regions. Each successive circle represents a differentially abundant taxonomic clades at phylum, class, order, family, genus and species level from the inner to outer rings. (B): Histograms of

the different abundant taxa based on the cutoff value of LDA score (log10) > 3.6 and P

< 0.05 between the FMT group from healthy controls and RA patients. The LDA scores of the FMT group from healthy controls were negative, while those of the FMT from RA patients were positive.


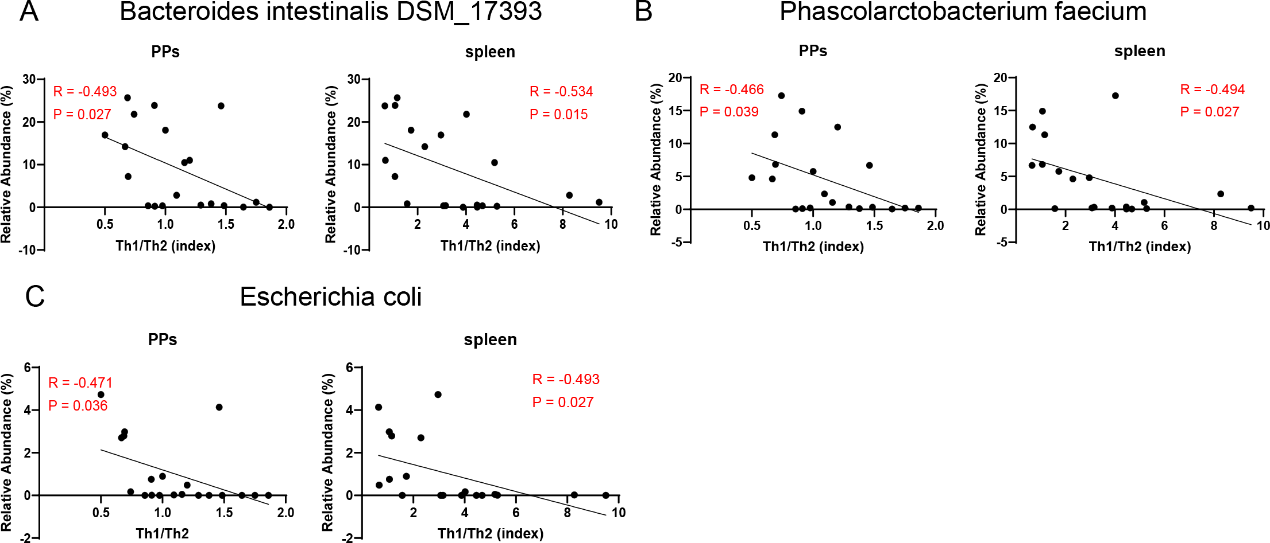


**Figure S7. Correlation of bacteria-species with Th1/Th2 cell from PPs and spleen.**

(A): *Bacteroides intestinalis DSM_17393*. (B): *Phascolarctobacterium faecium*. (C): *Escherichia coli*.

**Table S1. The characteristics of participants**

For age and BMI comparison, Mann-Whitney U-test was performed. For gender comparison, Fisher's exact test was performed. DAS28 = 28-Joint Disease Activity Score; CRP = C-reactive protein; ESR = erythrocyte sedimentation rate; CCP = cyclic citrullinated peptide.

**Table S2. Statistical analysis data of gut microbiota at genus level after FMT**

**Table S3. Statistical analysis data of gut microbiota at species level after FMT**
